# In this month’s Bulletin 

**DOI:** 10.2471/BLT.23.001023

**Published:** 2023-10-01

In the editorial section this month, Lisa M Askie et al. (618) call for papers on the impact of WHO’s normative and standard-setting function. Maria Eugenia Grillet (619) discusses what’s needed to sustain malaria elimination in the Region of the Americas.

Gary Humphreys and Lynn Eaton (622–623) report on the release of mosquito nets that use chlorfenapyr. Rosanna Peeling talks to Gary Humphreys (624–625) about the need for more use of new diagnostic tools.

## Bangladesh, Bhutan, Democratic People’s Republic of Korea, India, Indonesia, Maldives, Nepal, Sri Lanka, Thailand, Timor-Leste

### Ministers of Health in South-East Asia present the Paro Declaration 

Dechen Wangmo et al. (679–680) commit to action in mental health.

## Burkina Faso, Cameroon, Côte d’Ivoire, Democratic Republic of the Congo, Indonesia, Madagascar, Malaysia, Senegal

### Tracking pathogens from animal, human and environmental sources

Etienne Ruppé et al. (672–678) introduce a surveillance network for antimicrobial resistant pathogens.

## Uganda, United Republic of Tanzania

### Calculation of sustainable service provision

Ryan K McBain et al (626–636) determine the costs associated with provided HIV treatment.

## Global

### Accurate attribution of road traffic deaths 

Junjie Hua et al. (637–648) assess coding data quality in WHO’s mortality statistics.

### Listing communicable disease control targets

Laila Khawar et al. (649–665) review eradication and elimination goals.

### Towards more representative research 

Michelle R Kaufman et al. (666–671) explain why sex and gender need to be accounted for correctly.

